# 2-(4-Bromo­phen­yl)-2-oxoethyl 4-bromo­benzoate

**DOI:** 10.1107/S1600536811020654

**Published:** 2011-06-04

**Authors:** Hoong-Kun Fun, Suhana Arshad, B. Garudachari, Arun M. Isloor, M. N. Satyanarayan

**Affiliations:** a X-ray Crystallography Unit, School of Physics, Universiti Sains Malaysia, 11800 USM, Penang, Malaysia; b Organic Chemistry Division, Department of Chemistry, National Institute of Technology – Karnataka, Surathkal, Mangalore 575 025, India; c Department of Physics, National Institute of Technology – Karnataka, Surathkal, Mangalore 575 025, India

## Abstract

The asymmetric unit of the title compound, C_15_H_10_Br_2_O_3_, consists of three crystallographically independent mol­ecules (*A*, *B* and *C*). The phenyl rings in mol­ecules *A*, *B* and *C* make dihedral angles of 6.1 (3), 3.2 (2) and 54.6 (2)° to each other, respectively. In the crystal, mol­ecules are linked into two-dimensional layers parallel to the *ab* plane by inter­molecular C—H⋯O hydrogen bonds. The crystal structure is further stabilized by C—H⋯π inter­actions. The studied crystal is an inversion twin, the refined ratio of the twin components being 0.128 (8):0.872 (8).

## Related literature

For general background to phenacyl benzoates, see: Huang *et al.* (1996 ▶); Gandhi *et al.* (1995 ▶); Sheehan & Umezaw (1973 ▶); Ruzicka *et al.* (2002 ▶); Litera *et al.* (2006 ▶); Rather & Reid (1919 ▶). For the values of bond lengths, see: Allen *et al.* (1987 ▶). For stability of the temperature controller used for data collection, see: Cosier & Glazer (1986 ▶). For the synthetic procedure, see: Kelly & Howard (1932 ▶).
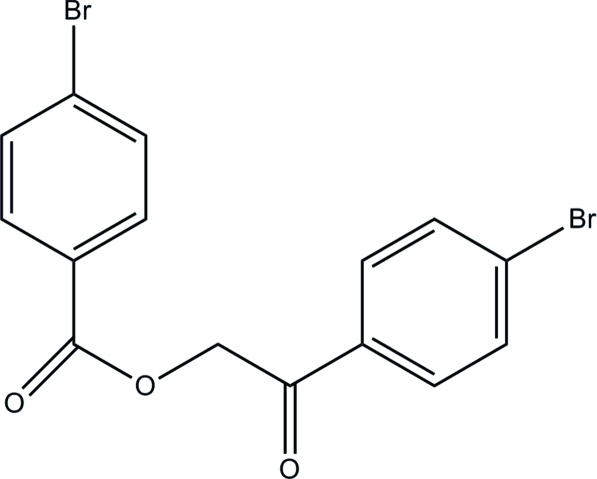


## Experimental

### 

#### Crystal data

C_15_H_10_Br_2_O_3_
*M* *_r_* = 398.05
Monoclinic, 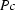

*a* = 11.0483 (3) Å
*b* = 5.9079 (1) Å
*c* = 33.8550 (8) Å
β = 108.802 (1)°
*V* = 2091.87 (8) Å^3^
*Z* = 6
Mo *K*α radiation
μ = 5.82 mm^−1^
*T* = 100 K
0.72 × 0.46 × 0.04 mm

#### Data collection

Bruker SMART APEXII CCD area-detector diffractometer
Absorption correction: multi-scan (*SADABS*; Bruker, 2009 ▶) *T*_min_ = 0.103, *T*_max_ = 0.809
43017 measured reflections
15850 independent reflections
11608 reflections with *I* > 2σ(*I*)
*R*_int_ = 0.050

#### Refinement

*R*[*F* ^2^ > 2σ(*F* ^2^)] = 0.047
*wR*(*F* ^2^) = 0.140
*S* = 0.98
15850 reflections
542 parameters
2 restraints
H-atom parameters constrained
Δρ_max_ = 1.39 e Å^−3^
Δρ_min_ = −1.13 e Å^−3^
Absolute structure: Flack (1983 ▶), 6614 Friedel pairs
Flack parameter: 0.128 (8)

### 

Data collection: *APEX2* (Bruker, 2009 ▶); cell refinement: *SAINT* (Bruker, 2009 ▶); data reduction: *SAINT*; program(s) used to solve structure: *SHELXTL* (Sheldrick, 2008 ▶); program(s) used to refine structure: *SHELXTL*; molecular graphics: *SHELXTL*; software used to prepare material for publication: *SHELXTL* and *PLATON* (Spek, 2009 ▶).

## Supplementary Material

Crystal structure: contains datablock(s) global, I. DOI: 10.1107/S1600536811020654/rz2603sup1.cif

Structure factors: contains datablock(s) I. DOI: 10.1107/S1600536811020654/rz2603Isup2.hkl

Supplementary material file. DOI: 10.1107/S1600536811020654/rz2603Isup3.cml

Additional supplementary materials:  crystallographic information; 3D view; checkCIF report

## Figures and Tables

**Table 1:** Hydrogen-bond geometry (Å, °) *Cg*1, *Cg*2, *Cg*3, *Cg*4, *Cg*5, and *Cg*6 are the centroids of the C1*A*–C6*A*, C10*A*–C15*A*, C1*B*–C6*B*, C10*B*–C15*B*, C1*C*–C6*C* and C10*C*–C15*C* benzene rings, respectively.

*D*—H⋯*A*	*D*—H	H⋯*A*	*D*⋯*A*	*D*—H⋯*A*
C8*A*—H8*AA*⋯O2*C*	0.99	2.39	3.041 (7)	122
C8*A*—H8*AB*⋯O2*B*	0.99	2.36	3.157 (6)	138
C5*B*—H5*BA*⋯O3*C*^i^	0.95	2.52	3.424 (6)	159
C2*C*—H2*CA*⋯O3*A*^ii^	0.95	2.35	3.093 (7)	135
C15*C*—H15*C*⋯O2*C*^iii^	0.95	2.52	3.408 (6)	155
C8*C*—H8*CB*⋯O3*B*^iv^	0.99	2.59	3.355 (6)	134
C1*B*—H1*BA*⋯*Cg*1	0.95	2.85	3.567 (6)	133
C14*B*—H14*B*⋯*Cg*2	0.95	2.78	3.498 (5)	133
C5*A*—H5*AA*⋯*Cg*3^ii^	0.95	2.75	3.401 (6)	126
C12*A*—H12*A*⋯*Cg*4^ii^	0.95	2.70	3.394 (6)	130
C5*C*—H5*CA*⋯*Cg*4^v^	0.95	2.94	3.691 (6)	137
C11*B*—H11*B*⋯*Cg*5^i^	0.95	2.93	3.596 (6)	128
C2*A*—H2*AA*⋯*Cg*6	0.95	2.83	3.425 (6)	122

Symmetry codes: (i) 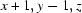; (ii) 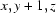; (iii) 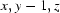; (iv) 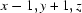; (v) 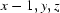.

**Fig. 1.**
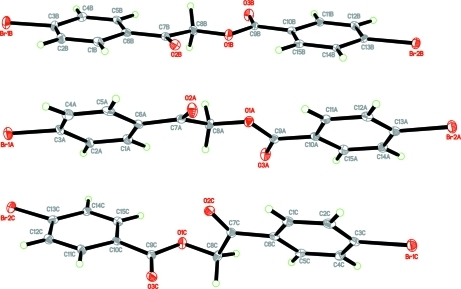
The molecular structure of the title compound, showing the three independent molecules with 30% probability displacement ellipsoids.

**Fig. 2.**
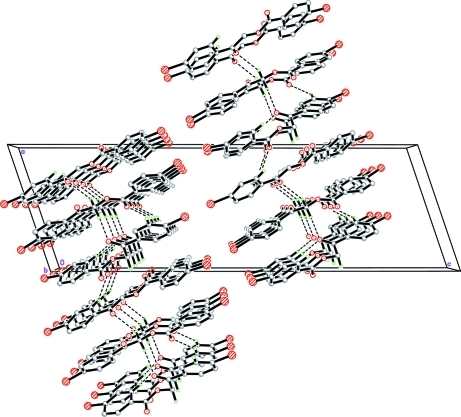
The crystal packing of the title compound viewed along the b axis. Dashed lines represent the hydrogen bonds.

## supplementary crystallographic information

### Comment

Phenacyl benzoates are very useful intermediates for the synthesis of biologically active oxazoles, imidazoles (Huang *et al.*, 1996) and benzoxazepine (Gandhi *et al.*, 1995). Phenacyl benzoates can be easily photolysed in completely neutral and mild conditions (Sheehan & Umezaw, 1973; Ruzicka *et al.*, 2002; Litera *et al.*, 2006). They are also used for identification of organic acids (Rather & Reid, 1919). Keeping this in view, we hereby report the crystal structure of 2-(4-bromophenyl)-2-oxoethyl 4-bromobenzoate of potential commercial importance.

The asymmetric unit of the tittle compound (Fig. 1), consists of three crystallographically independent molecules *A*, *B* and *C*. The phenyl rings (C1–C6, C10–C15) in molecules *A*, *B* and *C* make dihedral angles of 6.1 (3), 3.2 (2) and 54.6 (2)° to each other, respectively. The bond lengths (Allen *et al.*, 1987) and angles are within normal ranges. The crystal packing is shown in Fig. 2. The intermolecular C8A—H8AA···O2B and C8A—H8AB···O2C hydrogen bonds link molecule *A* with molecules *B* and *C*, respectively (Table 1). The molecules are linked into two-dimensional layers parallel to the *ab* plane by the intermolecular C5B—H5BA···O3C, C2C—H2CA···O3A, C15C—H15C···O2C and C8C—H8CB···O3B hydrogen bonds (Table 1). In addition, C—H···π interactions (Table 1) further stabilize the crystal structure.

### Experimental

The title compound was synthesized according to the method reported in the literature (Kelly & Howard, 1932). A mixture of 4-bromo benzoic acid (1.0 g, 0.0049 mol), sodium carbonate (0.579 g, 0.0054 mol) and 2-bromo-1-(4-bromophenyl)ethanone (1.50 g, 0.0054 mol) in dimethyl formamide (10 ml) was stirred at room temperature for 2 h. On cooling, the separated colourless block shaped crystals of 2-(4-bromophenyl)-2-oxoethyl 4-bromobenzoate were collected by filtration. The compound was recrystallized from ethanol. Yield: 1.80 g, 91.37%. M.p.: 407–408 K.

### Refinement

All H atoms were positioned geometrically [C–H = 0.95 or 0.99 Å] and refined using a riding model with *U*_iso_(H) = 1.2 *U*_eq_(C). The studied crystal is an inversion twin with the refined ratio of twin components being 0.128 (8):0.872 (8).

### Crystal data

**Table:**

C_15_H_10_Br_2_O_3_	*F*(000) = 1164
*M**_r_* = 398.05	*D*_x_ = 1.896 Mg m^−^^3^
Monoclinic, *P**c*	Mo *K*α radiation, λ = 0.71073 Å
Hall symbol: P -2yc	Cell parameters from 9925 reflections
*a* = 11.0483 (3) Å	θ = 2.5–33.5°
*b* = 5.9079 (1) Å	µ = 5.82 mm^−^^1^
*c* = 33.8550 (8) Å	*T* = 100 K
β = 108.802 (1)°	Block, colourless
*V* = 2091.87 (8) Å^3^	0.72 × 0.46 × 0.04 mm
*Z* = 6	

### Data collection

**Table:**

Bruker SMART APEXII CCD area-detector diffractometer	15850 independent reflections
Radiation source: fine-focus sealed tube	11608 reflections with *I* > 2σ(*I*)
graphite	*R*_int_ = 0.050
φ and ω scans	θ_max_ = 35.1°, θ_min_ = 1.3°
Absorption correction: multi-scan (*SADABS*; Bruker, 2009)	*h* = −16→17
*T*_min_ = 0.103, *T*_max_ = 0.809	*k* = −9→9
43017 measured reflections	*l* = −54→54

### Refinement

**Table:**

Refinement on *F*^2^	Secondary atom site location: difference Fourier map
Least-squares matrix: full	Hydrogen site location: inferred from neighbouring sites
*R*[*F*^2^ > 2σ(*F*^2^)] = 0.047	H-atom parameters constrained
*wR*(*F*^2^) = 0.140	*w* = 1/[σ^2^(*F*_o_^2^) + (0.0764*P*)^2^] where *P* = (*F*_o_^2^ + 2*F*_c_^2^)/3
*S* = 0.98	(Δ/σ)_max_ = 0.001
15850 reflections	Δρ_max_ = 1.39 e Å^−^^3^
542 parameters	Δρ_min_ = −1.13 e Å^−^^3^
2 restraints	Absolute structure: Flack (1983), 6614 Friedel pairs
Primary atom site location: structure-invariant direct methods	Flack parameter: 0.128 (8)

### Special details

**Table:**

Experimental. The crystal was placed in the cold stream of an Oxford Cryosystems Cobra open-flow nitrogen cryostat (Cosier & Glazer, 1986) operating at 100.0 (1) K.
Geometry. All e.s.d.'s (except the e.s.d. in the dihedral angle between two l.s. planes) are estimated using the full covariance matrix. The cell e.s.d.'s are taken into account individually in the estimation of e.s.d.'s in distances, angles and torsion angles; correlations between e.s.d.'s in cell parameters are only used when they are defined by crystal symmetry. An approximate (isotropic) treatment of cell e.s.d.'s is used for estimating e.s.d.'s involving l.s. planes.
Refinement. Refinement of *F*^2^ against ALL reflections. The weighted *R*-factor *wR* and goodness of fit *S* are based on *F*^2^, conventional *R*-factors *R* are based on *F*, with *F* set to zero for negative *F*^2^. The threshold expression of *F*^2^ > σ(*F*^2^) is used only for calculating *R*-factors(gt) *etc*. and is not relevant to the choice of reflections for refinement. *R*-factors based on *F*^2^ are statistically about twice as large as those based on *F*, and *R*- factors based on ALL data will be even larger.

### Fractional atomic coordinates and isotropic or equivalent isotropic displacement parameters (Å^2^)

**Table:**

	*x*	*y*	*z*	*U*_iso_*/*U*_eq_	
Br1A	0.18159 (5)	0.60252 (8)	−0.036383 (16)	0.02639 (11)	
Br2A	0.82436 (5)	1.43958 (8)	0.402087 (16)	0.02523 (11)	
O1A	0.5680 (3)	0.9675 (6)	0.21033 (11)	0.0223 (7)	
O2A	0.5031 (4)	1.1995 (6)	0.13989 (11)	0.0278 (8)	
O3A	0.5118 (4)	0.6926 (6)	0.24696 (11)	0.0259 (7)	
C1A	0.3424 (5)	0.6872 (7)	0.09101 (15)	0.0199 (9)	
H1AA	0.3506	0.5992	0.1152	0.024*	
C2A	0.2759 (5)	0.6015 (7)	0.05192 (15)	0.0188 (9)	
H2AA	0.2362	0.4572	0.0493	0.023*	
C3A	0.2679 (5)	0.7291 (8)	0.01649 (15)	0.0197 (9)	
C4A	0.3201 (5)	0.9442 (8)	0.01975 (17)	0.0235 (10)	
H4AA	0.3104	1.0329	−0.0045	0.028*	
C5A	0.3873 (5)	1.0282 (8)	0.05928 (17)	0.0216 (9)	
H5AA	0.4265	1.1730	0.0619	0.026*	
C6A	0.3973 (4)	0.9012 (7)	0.09495 (15)	0.0175 (8)	
C7A	0.4720 (4)	1.0006 (8)	0.13636 (15)	0.0185 (8)	
C8A	0.5071 (5)	0.8420 (7)	0.17317 (14)	0.0196 (8)	
H8AA	0.4292	0.7676	0.1753	0.024*	
H8AB	0.5656	0.7232	0.1694	0.024*	
C9A	0.5619 (4)	0.8729 (7)	0.24557 (14)	0.0167 (8)	
C10A	0.6243 (4)	1.0141 (7)	0.28312 (14)	0.0162 (8)	
C11A	0.6732 (4)	1.2286 (7)	0.27958 (14)	0.0172 (8)	
H11A	0.6661	1.2880	0.2528	0.021*	
C12A	0.7326 (5)	1.3554 (8)	0.31541 (16)	0.0207 (9)	
H12A	0.7660	1.5015	0.3134	0.025*	
C13A	0.7417 (5)	1.2645 (7)	0.35375 (15)	0.0195 (9)	
C14A	0.6945 (5)	1.0528 (8)	0.35786 (15)	0.0210 (9)	
H14A	0.7026	0.9935	0.3847	0.025*	
C15A	0.6352 (5)	0.9284 (7)	0.32225 (15)	0.0201 (9)	
H15A	0.6016	0.7829	0.3246	0.024*	
Br1B	0.53003 (5)	0.07466 (9)	−0.054856 (16)	0.02998 (12)	
Br2B	1.07283 (4)	0.92900 (7)	0.388119 (15)	0.02288 (10)	
O1B	0.8378 (3)	0.4512 (5)	0.19518 (10)	0.0210 (7)	
O2B	0.7000 (3)	0.6665 (5)	0.12859 (11)	0.0227 (7)	
O3B	0.9667 (4)	0.1658 (6)	0.22740 (11)	0.0265 (7)	
C1B	0.5978 (5)	0.4985 (8)	0.04755 (16)	0.0208 (9)	
H1BA	0.5712	0.6439	0.0535	0.025*	
C2B	0.5533 (5)	0.4158 (7)	0.00704 (15)	0.0214 (9)	
H2BA	0.4975	0.5033	−0.0149	0.026*	
C3B	0.5927 (5)	0.2018 (8)	−0.00054 (14)	0.0205 (9)	
C4B	0.6760 (5)	0.0715 (7)	0.03099 (16)	0.0208 (9)	
H4BA	0.7024	−0.0737	0.0249	0.025*	
C5B	0.7197 (4)	0.1557 (8)	0.07135 (15)	0.0192 (8)	
H5BA	0.7757	0.0680	0.0932	0.023*	
C6B	0.6808 (4)	0.3716 (7)	0.07970 (15)	0.0177 (8)	
C7B	0.7254 (4)	0.4719 (7)	0.12233 (14)	0.0171 (8)	
C8B	0.8037 (5)	0.3228 (8)	0.15763 (14)	0.0210 (9)	
H8BA	0.7533	0.1884	0.1602	0.025*	
H8BB	0.8817	0.2704	0.1520	0.025*	
C9B	0.9212 (4)	0.3521 (7)	0.22846 (13)	0.0169 (8)	
C10B	0.9545 (4)	0.4987 (8)	0.26645 (15)	0.0179 (8)	
C11B	1.0415 (5)	0.4121 (7)	0.30300 (15)	0.0187 (9)	
H11B	1.0774	0.2661	0.3028	0.022*	
C12B	1.0756 (5)	0.5370 (8)	0.33938 (16)	0.0205 (9)	
H12B	1.1340	0.4776	0.3644	0.025*	
C13B	1.0231 (5)	0.7509 (7)	0.33885 (14)	0.0177 (8)	
C14B	0.9370 (4)	0.8397 (7)	0.30301 (14)	0.0160 (8)	
H14B	0.9018	0.9861	0.3033	0.019*	
C15B	0.9024 (4)	0.7123 (7)	0.26641 (14)	0.0179 (8)	
H15B	0.8435	0.7715	0.2415	0.021*	
Br1C	0.43511 (5)	1.43292 (8)	0.377352 (16)	0.02489 (11)	
Br2C	−0.05174 (5)	0.07320 (8)	−0.018770 (15)	0.02514 (11)	
O1C	0.1123 (4)	0.5932 (5)	0.17085 (11)	0.0219 (7)	
O2C	0.2506 (3)	0.9773 (6)	0.18127 (11)	0.0228 (7)	
O3C	−0.0252 (3)	0.8552 (6)	0.13377 (11)	0.0226 (7)	
C1C	0.3297 (4)	1.2443 (7)	0.25390 (15)	0.0195 (8)	
H1CA	0.3358	1.3074	0.2288	0.023*	
C2C	0.3796 (5)	1.3622 (8)	0.29096 (17)	0.0222 (9)	
H2CA	0.4201	1.5046	0.2916	0.027*	
C3C	0.3688 (4)	1.2664 (7)	0.32701 (15)	0.0184 (8)	
C4C	0.3121 (5)	1.0557 (7)	0.32713 (16)	0.0206 (9)	
H4CA	0.3079	0.9920	0.3524	0.025*	
C5C	0.2617 (5)	0.9405 (7)	0.28960 (15)	0.0185 (8)	
H5CA	0.2213	0.7980	0.2890	0.022*	
C6C	0.2706 (4)	1.0347 (7)	0.25272 (15)	0.0177 (8)	
C7C	0.2193 (4)	0.9178 (7)	0.21081 (14)	0.0178 (8)	
C8C	0.1289 (5)	0.7231 (7)	0.20783 (14)	0.0213 (9)	
H8CA	0.1628	0.6243	0.2326	0.026*	
H8CB	0.0449	0.7822	0.2077	0.026*	
C9C	0.0324 (4)	0.6806 (7)	0.13537 (13)	0.0180 (8)	
C10C	0.0191 (4)	0.5316 (7)	0.09875 (14)	0.0163 (8)	
C11C	−0.0566 (5)	0.6070 (7)	0.05980 (15)	0.0189 (9)	
H11C	−0.0946	0.7528	0.0572	0.023*	
C12C	−0.0778 (5)	0.4725 (8)	0.02446 (15)	0.0210 (9)	
H12C	−0.1305	0.5242	−0.0021	0.025*	
C13C	−0.0203 (5)	0.2613 (7)	0.02892 (15)	0.0209 (9)	
C14C	0.0574 (5)	0.1815 (7)	0.06756 (14)	0.0188 (8)	
H14C	0.0963	0.0365	0.0700	0.023*	
C15C	0.0765 (4)	0.3188 (7)	0.10221 (15)	0.0185 (9)	
H15C	0.1294	0.2673	0.1287	0.022*	

### Atomic displacement parameters (Å^2^)

**Table:**

	*U*^11^	*U*^22^	*U*^33^	*U*^12^	*U*^13^	*U*^23^
Br1A	0.0275 (3)	0.0295 (2)	0.0182 (2)	−0.0036 (2)	0.0018 (2)	−0.00314 (18)
Br2A	0.0262 (3)	0.0274 (2)	0.0195 (2)	−0.0049 (2)	0.0038 (2)	−0.00427 (18)
O1A	0.0243 (18)	0.0250 (16)	0.0185 (16)	−0.0052 (13)	0.0083 (14)	−0.0009 (12)
O2A	0.036 (2)	0.0218 (16)	0.0214 (17)	−0.0107 (15)	0.0038 (15)	−0.0034 (13)
O3A	0.036 (2)	0.0206 (15)	0.0210 (16)	−0.0059 (14)	0.0085 (15)	0.0007 (12)
C1A	0.019 (2)	0.0192 (19)	0.020 (2)	−0.0022 (17)	0.0044 (18)	0.0024 (15)
C2A	0.017 (2)	0.0210 (19)	0.016 (2)	−0.0030 (16)	0.0025 (17)	0.0029 (15)
C3A	0.015 (2)	0.023 (2)	0.018 (2)	−0.0028 (16)	0.0009 (17)	−0.0020 (16)
C4A	0.022 (2)	0.023 (2)	0.026 (2)	0.0025 (18)	0.008 (2)	0.0072 (17)
C5A	0.015 (2)	0.0174 (19)	0.032 (3)	−0.0012 (16)	0.008 (2)	0.0024 (17)
C6A	0.014 (2)	0.0205 (19)	0.018 (2)	0.0003 (15)	0.0047 (17)	0.0018 (15)
C7A	0.015 (2)	0.0207 (18)	0.021 (2)	−0.0017 (17)	0.0074 (17)	−0.0012 (16)
C8A	0.025 (2)	0.0185 (19)	0.0165 (19)	−0.0017 (17)	0.0086 (18)	−0.0034 (15)
C9A	0.015 (2)	0.0181 (18)	0.0169 (19)	0.0030 (15)	0.0045 (17)	0.0017 (14)
C10A	0.0127 (19)	0.0179 (18)	0.018 (2)	0.0021 (15)	0.0044 (16)	0.0038 (15)
C11A	0.016 (2)	0.0197 (18)	0.0164 (19)	0.0037 (16)	0.0062 (17)	0.0050 (15)
C12A	0.017 (2)	0.0196 (19)	0.027 (2)	−0.0009 (17)	0.0081 (19)	0.0021 (17)
C13A	0.016 (2)	0.021 (2)	0.022 (2)	0.0027 (17)	0.0070 (18)	0.0039 (16)
C14A	0.019 (2)	0.025 (2)	0.019 (2)	−0.0023 (17)	0.0068 (18)	0.0023 (16)
C15A	0.020 (2)	0.0189 (19)	0.021 (2)	0.0010 (16)	0.0066 (18)	0.0040 (15)
Br1B	0.0351 (3)	0.0346 (3)	0.0171 (2)	0.0034 (2)	0.0040 (2)	−0.00323 (18)
Br2B	0.0263 (3)	0.0243 (2)	0.0178 (2)	−0.00191 (18)	0.00666 (19)	−0.00410 (16)
O1B	0.0251 (18)	0.0201 (15)	0.0160 (15)	0.0032 (13)	0.0039 (14)	−0.0018 (11)
O2B	0.0229 (17)	0.0204 (15)	0.0247 (17)	0.0002 (13)	0.0073 (14)	−0.0012 (13)
O3B	0.033 (2)	0.0202 (15)	0.0232 (17)	0.0077 (14)	0.0052 (15)	−0.0001 (13)
C1B	0.018 (2)	0.0156 (17)	0.028 (2)	0.0037 (16)	0.0065 (19)	0.0037 (16)
C2B	0.021 (2)	0.023 (2)	0.018 (2)	0.0008 (17)	0.0031 (18)	0.0022 (16)
C3B	0.024 (2)	0.023 (2)	0.0139 (19)	0.0000 (18)	0.0054 (18)	0.0002 (15)
C4B	0.023 (2)	0.0164 (19)	0.025 (2)	0.0001 (16)	0.010 (2)	−0.0014 (15)
C5B	0.014 (2)	0.0196 (19)	0.023 (2)	0.0016 (16)	0.0039 (18)	0.0018 (16)
C6B	0.014 (2)	0.0167 (17)	0.022 (2)	0.0007 (15)	0.0049 (17)	0.0042 (15)
C7B	0.015 (2)	0.0170 (18)	0.020 (2)	0.0005 (15)	0.0065 (17)	0.0022 (15)
C8B	0.020 (2)	0.0216 (19)	0.020 (2)	−0.0006 (17)	0.0041 (18)	−0.0047 (16)
C9B	0.016 (2)	0.0214 (19)	0.0130 (18)	−0.0034 (16)	0.0047 (16)	−0.0007 (15)
C10B	0.016 (2)	0.0196 (18)	0.020 (2)	−0.0002 (16)	0.0085 (17)	−0.0002 (16)
C11B	0.016 (2)	0.0194 (19)	0.020 (2)	0.0013 (16)	0.0055 (18)	−0.0004 (15)
C12B	0.017 (2)	0.021 (2)	0.022 (2)	−0.0007 (17)	0.0038 (18)	0.0028 (16)
C13B	0.018 (2)	0.0217 (19)	0.016 (2)	−0.0059 (16)	0.0094 (17)	−0.0043 (15)
C14B	0.013 (2)	0.0193 (18)	0.0159 (19)	−0.0026 (15)	0.0051 (16)	−0.0025 (14)
C15B	0.015 (2)	0.0168 (18)	0.023 (2)	0.0007 (15)	0.0079 (18)	0.0007 (15)
Br1C	0.0246 (3)	0.0249 (2)	0.0225 (2)	−0.00107 (19)	0.00391 (19)	−0.00451 (18)
Br2C	0.0315 (3)	0.0247 (2)	0.0194 (2)	−0.0009 (2)	0.0084 (2)	−0.00358 (17)
O1C	0.0286 (19)	0.0186 (15)	0.0163 (15)	0.0008 (13)	0.0042 (14)	−0.0003 (11)
O2C	0.0203 (17)	0.0306 (17)	0.0189 (16)	−0.0016 (14)	0.0082 (14)	0.0037 (13)
O3C	0.0191 (17)	0.0242 (15)	0.0227 (17)	0.0028 (13)	0.0045 (14)	−0.0009 (13)
C1C	0.017 (2)	0.0185 (19)	0.023 (2)	0.0004 (16)	0.0076 (18)	0.0035 (16)
C2C	0.017 (2)	0.0168 (18)	0.034 (3)	−0.0022 (17)	0.010 (2)	−0.0003 (17)
C3C	0.012 (2)	0.0192 (19)	0.023 (2)	−0.0005 (15)	0.0036 (17)	−0.0003 (16)
C4C	0.022 (2)	0.0190 (19)	0.023 (2)	0.0024 (17)	0.0091 (19)	0.0007 (16)
C5C	0.017 (2)	0.0183 (19)	0.023 (2)	0.0022 (16)	0.0095 (18)	0.0010 (15)
C6C	0.0113 (19)	0.0185 (19)	0.023 (2)	0.0027 (15)	0.0059 (17)	0.0017 (15)
C7C	0.016 (2)	0.0177 (18)	0.018 (2)	0.0038 (16)	0.0031 (17)	0.0003 (15)
C8C	0.030 (3)	0.0175 (19)	0.019 (2)	−0.0013 (17)	0.0105 (19)	−0.0002 (15)
C9C	0.018 (2)	0.0190 (18)	0.0172 (19)	−0.0032 (16)	0.0064 (17)	0.0019 (15)
C10C	0.013 (2)	0.0185 (18)	0.0164 (19)	−0.0017 (15)	0.0040 (16)	0.0007 (14)
C11C	0.017 (2)	0.0188 (19)	0.019 (2)	0.0008 (16)	0.0029 (18)	0.0036 (15)
C12C	0.021 (2)	0.021 (2)	0.021 (2)	0.0010 (17)	0.0049 (18)	0.0025 (16)
C13C	0.018 (2)	0.0196 (19)	0.027 (2)	−0.0040 (17)	0.0093 (19)	−0.0075 (17)
C14C	0.020 (2)	0.0185 (19)	0.019 (2)	−0.0001 (16)	0.0079 (18)	0.0005 (15)
C15C	0.018 (2)	0.0147 (17)	0.022 (2)	−0.0007 (16)	0.0058 (18)	0.0049 (15)

### Geometric parameters (Å, °)

**Table:**

Br1A—C3A	1.890 (5)	C6B—C7B	1.489 (6)
Br2A—C13A	1.902 (5)	C7B—C8B	1.512 (6)
O1A—C9A	1.339 (5)	C8B—H8BA	0.9900
O1A—C8A	1.428 (5)	C8B—H8BB	0.9900
O2A—C7A	1.220 (6)	C9B—C10B	1.495 (6)
O3A—C9A	1.208 (5)	C10B—C15B	1.386 (6)
C1A—C2A	1.386 (7)	C10B—C11B	1.397 (7)
C1A—C6A	1.390 (6)	C11B—C12B	1.380 (7)
C1A—H1AA	0.9500	C11B—H11B	0.9500
C2A—C3A	1.395 (6)	C12B—C13B	1.388 (6)
C2A—H2AA	0.9500	C12B—H12B	0.9500
C3A—C4A	1.385 (6)	C13B—C14B	1.381 (6)
C4A—C5A	1.396 (7)	C14B—C15B	1.394 (6)
C4A—H4AA	0.9500	C14B—H14B	0.9500
C5A—C6A	1.396 (7)	C15B—H15B	0.9500
C5A—H5AA	0.9500	Br1C—C3C	1.898 (5)
C6A—C7A	1.499 (7)	Br2C—C13C	1.898 (4)
C7A—C8A	1.506 (7)	O1C—C9C	1.344 (5)
C8A—H8AA	0.9900	O1C—C8C	1.429 (5)
C8A—H8AB	0.9900	O2C—C7C	1.211 (5)
C9A—C10A	1.491 (6)	O3C—C9C	1.204 (5)
C10A—C15A	1.387 (6)	C1C—C2C	1.385 (7)
C10A—C11A	1.398 (6)	C1C—C6C	1.395 (6)
C11A—C12A	1.396 (7)	C1C—H1CA	0.9500
C11A—H11A	0.9500	C2C—C3C	1.385 (7)
C12A—C13A	1.378 (7)	C2C—H2CA	0.9500
C12A—H12A	0.9500	C3C—C4C	1.395 (6)
C13A—C14A	1.379 (6)	C4C—C5C	1.390 (7)
C14A—C15A	1.384 (7)	C4C—H4CA	0.9500
C14A—H14A	0.9500	C5C—C6C	1.399 (6)
C15A—H15A	0.9500	C5C—H5CA	0.9500
Br1B—C3B	1.898 (5)	C6C—C7C	1.514 (6)
Br2B—C13B	1.898 (4)	C7C—C8C	1.505 (6)
O1B—C9B	1.339 (6)	C8C—H8CA	0.9900
O1B—C8B	1.423 (5)	C8C—H8CB	0.9900
O2B—C7B	1.218 (5)	C9C—C10C	1.489 (6)
O3B—C9B	1.215 (5)	C10C—C11C	1.388 (6)
C1B—C2B	1.388 (7)	C10C—C15C	1.396 (6)
C1B—C6B	1.395 (6)	C11C—C12C	1.392 (7)
C1B—H1BA	0.9500	C11C—H11C	0.9500
C2B—C3B	1.388 (6)	C12C—C13C	1.386 (6)
C2B—H2BA	0.9500	C12C—H12C	0.9500
C3B—C4B	1.395 (7)	C13C—C14C	1.395 (7)
C4B—C5B	1.387 (7)	C14C—C15C	1.385 (6)
C4B—H4BA	0.9500	C14C—H14C	0.9500
C5B—C6B	1.404 (6)	C15C—H15C	0.9500
C5B—H5BA	0.9500		
			
C9A—O1A—C8A	115.1 (4)	O1B—C8B—H8BB	110.0
C2A—C1A—C6A	120.3 (4)	C7B—C8B—H8BB	110.0
C2A—C1A—H1AA	119.9	H8BA—C8B—H8BB	108.4
C6A—C1A—H1AA	119.9	O3B—C9B—O1B	123.4 (4)
C1A—C2A—C3A	119.4 (4)	O3B—C9B—C10B	124.1 (4)
C1A—C2A—H2AA	120.3	O1B—C9B—C10B	112.5 (4)
C3A—C2A—H2AA	120.3	C15B—C10B—C11B	120.1 (4)
C4A—C3A—C2A	121.2 (4)	C15B—C10B—C9B	122.6 (4)
C4A—C3A—Br1A	120.6 (4)	C11B—C10B—C9B	117.3 (4)
C2A—C3A—Br1A	118.2 (3)	C12B—C11B—C10B	120.4 (4)
C3A—C4A—C5A	118.8 (4)	C12B—C11B—H11B	119.8
C3A—C4A—H4AA	120.6	C10B—C11B—H11B	119.8
C5A—C4A—H4AA	120.6	C11B—C12B—C13B	118.9 (5)
C6A—C5A—C4A	120.5 (4)	C11B—C12B—H12B	120.6
C6A—C5A—H5AA	119.7	C13B—C12B—H12B	120.6
C4A—C5A—H5AA	119.7	C14B—C13B—C12B	121.6 (4)
C1A—C6A—C5A	119.7 (4)	C14B—C13B—Br2B	118.9 (3)
C1A—C6A—C7A	122.5 (4)	C12B—C13B—Br2B	119.5 (4)
C5A—C6A—C7A	117.7 (4)	C13B—C14B—C15B	119.3 (4)
O2A—C7A—C6A	121.5 (4)	C13B—C14B—H14B	120.3
O2A—C7A—C8A	121.9 (4)	C15B—C14B—H14B	120.3
C6A—C7A—C8A	116.6 (4)	C10B—C15B—C14B	119.7 (4)
O1A—C8A—C7A	109.2 (4)	C10B—C15B—H15B	120.1
O1A—C8A—H8AA	109.8	C14B—C15B—H15B	120.1
C7A—C8A—H8AA	109.8	C9C—O1C—C8C	116.0 (3)
O1A—C8A—H8AB	109.8	C2C—C1C—C6C	121.2 (4)
C7A—C8A—H8AB	109.8	C2C—C1C—H1CA	119.4
H8AA—C8A—H8AB	108.3	C6C—C1C—H1CA	119.4
O3A—C9A—O1A	123.9 (4)	C1C—C2C—C3C	118.1 (4)
O3A—C9A—C10A	123.5 (4)	C1C—C2C—H2CA	121.0
O1A—C9A—C10A	112.6 (4)	C3C—C2C—H2CA	121.0
C15A—C10A—C11A	119.7 (4)	C2C—C3C—C4C	122.3 (4)
C15A—C10A—C9A	119.0 (4)	C2C—C3C—Br1C	117.5 (3)
C11A—C10A—C9A	121.3 (4)	C4C—C3C—Br1C	120.2 (4)
C12A—C11A—C10A	119.9 (4)	C5C—C4C—C3C	118.8 (4)
C12A—C11A—H11A	120.1	C5C—C4C—H4CA	120.6
C10A—C11A—H11A	120.1	C3C—C4C—H4CA	120.6
C13A—C12A—C11A	118.7 (4)	C4C—C5C—C6C	119.9 (4)
C13A—C12A—H12A	120.6	C4C—C5C—H5CA	120.0
C11A—C12A—H12A	120.6	C6C—C5C—H5CA	120.0
C12A—C13A—C14A	122.2 (5)	C1C—C6C—C5C	119.6 (4)
C12A—C13A—Br2A	117.9 (4)	C1C—C6C—C7C	117.5 (4)
C14A—C13A—Br2A	119.9 (4)	C5C—C6C—C7C	122.8 (4)
C13A—C14A—C15A	118.8 (4)	O2C—C7C—C8C	121.8 (4)
C13A—C14A—H14A	120.6	O2C—C7C—C6C	121.4 (4)
C15A—C14A—H14A	120.6	C8C—C7C—C6C	116.8 (4)
C14A—C15A—C10A	120.6 (4)	O1C—C8C—C7C	111.3 (4)
C14A—C15A—H15A	119.7	O1C—C8C—H8CA	109.4
C10A—C15A—H15A	119.7	C7C—C8C—H8CA	109.4
C9B—O1B—C8B	115.5 (3)	O1C—C8C—H8CB	109.4
C2B—C1B—C6B	121.1 (4)	C7C—C8C—H8CB	109.4
C2B—C1B—H1BA	119.5	H8CA—C8C—H8CB	108.0
C6B—C1B—H1BA	119.5	O3C—C9C—O1C	123.7 (4)
C1B—C2B—C3B	118.1 (4)	O3C—C9C—C10C	124.1 (4)
C1B—C2B—H2BA	121.0	O1C—C9C—C10C	112.2 (4)
C3B—C2B—H2BA	121.0	C11C—C10C—C15C	119.1 (4)
C2B—C3B—C4B	122.0 (4)	C11C—C10C—C9C	118.0 (4)
C2B—C3B—Br1B	120.1 (4)	C15C—C10C—C9C	122.9 (4)
C4B—C3B—Br1B	117.9 (3)	C10C—C11C—C12C	121.1 (4)
C5B—C4B—C3B	119.4 (4)	C10C—C11C—H11C	119.4
C5B—C4B—H4BA	120.3	C12C—C11C—H11C	119.4
C3B—C4B—H4BA	120.3	C13C—C12C—C11C	118.4 (4)
C4B—C5B—C6B	119.5 (4)	C13C—C12C—H12C	120.8
C4B—C5B—H5BA	120.2	C11C—C12C—H12C	120.8
C6B—C5B—H5BA	120.2	C12C—C13C—C14C	121.9 (4)
C1B—C6B—C5B	119.9 (4)	C12C—C13C—Br2C	118.9 (4)
C1B—C6B—C7B	118.1 (4)	C14C—C13C—Br2C	119.2 (3)
C5B—C6B—C7B	122.0 (4)	C15C—C14C—C13C	118.4 (4)
O2B—C7B—C6B	121.4 (4)	C15C—C14C—H14C	120.8
O2B—C7B—C8B	121.2 (4)	C13C—C14C—H14C	120.8
C6B—C7B—C8B	117.4 (4)	C14C—C15C—C10C	121.0 (4)
O1B—C8B—C7B	108.3 (3)	C14C—C15C—H15C	119.5
O1B—C8B—H8BA	110.0	C10C—C15C—H15C	119.5
C7B—C8B—H8BA	110.0		
			
C6A—C1A—C2A—C3A	2.0 (7)	C8B—O1B—C9B—O3B	0.5 (6)
C1A—C2A—C3A—C4A	−3.0 (7)	C8B—O1B—C9B—C10B	178.8 (4)
C1A—C2A—C3A—Br1A	177.8 (4)	O3B—C9B—C10B—C15B	178.8 (4)
C2A—C3A—C4A—C5A	3.3 (7)	O1B—C9B—C10B—C15B	0.5 (6)
Br1A—C3A—C4A—C5A	−177.6 (4)	O3B—C9B—C10B—C11B	−1.4 (7)
C3A—C4A—C5A—C6A	−2.5 (7)	O1B—C9B—C10B—C11B	−179.7 (4)
C2A—C1A—C6A—C5A	−1.2 (7)	C15B—C10B—C11B—C12B	0.5 (7)
C2A—C1A—C6A—C7A	−179.0 (4)	C9B—C10B—C11B—C12B	−179.3 (4)
C4A—C5A—C6A—C1A	1.5 (7)	C10B—C11B—C12B—C13B	−0.7 (7)
C4A—C5A—C6A—C7A	179.3 (4)	C11B—C12B—C13B—C14B	0.7 (7)
C1A—C6A—C7A—O2A	−169.0 (5)	C11B—C12B—C13B—Br2B	−178.1 (4)
C5A—C6A—C7A—O2A	13.2 (7)	C12B—C13B—C14B—C15B	−0.4 (7)
C1A—C6A—C7A—C8A	11.3 (6)	Br2B—C13B—C14B—C15B	178.4 (3)
C5A—C6A—C7A—C8A	−166.4 (4)	C11B—C10B—C15B—C14B	−0.2 (6)
C9A—O1A—C8A—C7A	156.4 (4)	C9B—C10B—C15B—C14B	179.6 (4)
O2A—C7A—C8A—O1A	5.2 (6)	C13B—C14B—C15B—C10B	0.1 (6)
C6A—C7A—C8A—O1A	−175.2 (4)	C6C—C1C—C2C—C3C	−0.3 (7)
C8A—O1A—C9A—O3A	1.6 (6)	C1C—C2C—C3C—C4C	1.2 (7)
C8A—O1A—C9A—C10A	−178.6 (4)	C1C—C2C—C3C—Br1C	−178.9 (3)
O3A—C9A—C10A—C15A	5.4 (7)	C2C—C3C—C4C—C5C	−1.7 (7)
O1A—C9A—C10A—C15A	−174.4 (4)	Br1C—C3C—C4C—C5C	178.5 (3)
O3A—C9A—C10A—C11A	−175.5 (4)	C3C—C4C—C5C—C6C	1.1 (7)
O1A—C9A—C10A—C11A	4.7 (6)	C2C—C1C—C6C—C5C	−0.2 (7)
C15A—C10A—C11A—C12A	−0.1 (7)	C2C—C1C—C6C—C7C	−179.8 (4)
C9A—C10A—C11A—C12A	−179.2 (4)	C4C—C5C—C6C—C1C	−0.2 (7)
C10A—C11A—C12A—C13A	0.2 (7)	C4C—C5C—C6C—C7C	179.3 (4)
C11A—C12A—C13A—C14A	0.1 (7)	C1C—C6C—C7C—O2C	15.1 (6)
C11A—C12A—C13A—Br2A	179.5 (3)	C5C—C6C—C7C—O2C	−164.4 (4)
C12A—C13A—C14A—C15A	−0.5 (7)	C1C—C6C—C7C—C8C	−165.6 (4)
Br2A—C13A—C14A—C15A	−179.8 (4)	C5C—C6C—C7C—C8C	14.9 (6)
C13A—C14A—C15A—C10A	0.5 (7)	C9C—O1C—C8C—C7C	−78.8 (5)
C11A—C10A—C15A—C14A	−0.3 (7)	O2C—C7C—C8C—O1C	13.7 (6)
C9A—C10A—C15A—C14A	178.8 (4)	C6C—C7C—C8C—O1C	−165.6 (4)
C6B—C1B—C2B—C3B	0.7 (7)	C8C—O1C—C9C—O3C	−1.3 (6)
C1B—C2B—C3B—C4B	−0.9 (7)	C8C—O1C—C9C—C10C	−178.8 (4)
C1B—C2B—C3B—Br1B	177.6 (4)	O3C—C9C—C10C—C11C	5.2 (7)
C2B—C3B—C4B—C5B	0.9 (7)	O1C—C9C—C10C—C11C	−177.3 (4)
Br1B—C3B—C4B—C5B	−177.6 (4)	O3C—C9C—C10C—C15C	−173.0 (4)
C3B—C4B—C5B—C6B	−0.8 (7)	O1C—C9C—C10C—C15C	4.5 (6)
C2B—C1B—C6B—C5B	−0.6 (7)	C15C—C10C—C11C—C12C	1.1 (7)
C2B—C1B—C6B—C7B	179.5 (4)	C9C—C10C—C11C—C12C	−177.2 (4)
C4B—C5B—C6B—C1B	0.6 (7)	C10C—C11C—C12C—C13C	−0.6 (7)
C4B—C5B—C6B—C7B	−179.5 (4)	C11C—C12C—C13C—C14C	0.0 (7)
C1B—C6B—C7B—O2B	−6.5 (7)	C11C—C12C—C13C—Br2C	178.5 (4)
C5B—C6B—C7B—O2B	173.6 (4)	C12C—C13C—C14C—C15C	0.2 (7)
C1B—C6B—C7B—C8B	173.6 (4)	Br2C—C13C—C14C—C15C	−178.3 (3)
C5B—C6B—C7B—C8B	−6.3 (6)	C13C—C14C—C15C—C10C	0.3 (7)
C9B—O1B—C8B—C7B	−171.8 (4)	C11C—C10C—C15C—C14C	−0.9 (7)
O2B—C7B—C8B—O1B	−0.7 (6)	C9C—C10C—C15C—C14C	177.3 (4)
C6B—C7B—C8B—O1B	179.2 (4)		

### Hydrogen-bond geometry (Å, °)

**Table:**

Cg1, Cg2, Cg3, Cg4, Cg5, and Cg6 are the centroids of the C1A–C6A, C10A–C15A, C1B–C6B, C10B–C15B, C1C–C6C and C10C–C15C benzene rings, respectively.

**Table:**

*D*—H···*A*	*D*—H	H···*A*	*D*···*A*	*D*—H···*A*
C8A—H8AA···O2C	0.99	2.39	3.041 (7)	122.
C8A—H8AB···O2B	0.99	2.36	3.157 (6)	138.
C5B—H5BA···O3C^i^	0.95	2.52	3.424 (6)	159.
C2C—H2CA···O3A^ii^	0.95	2.35	3.093 (7)	135.
C15C—H15C···O2C^iii^	0.95	2.52	3.408 (6)	155.
C8C—H8CB···O3B^iv^	0.99	2.59	3.355 (6)	134.
C1B—H1BA···Cg1	0.95	2.85	3.567 (6)	133.
C14B—H14B···Cg2	0.95	2.78	3.498 (5)	133.
C5A—H5AA···Cg3^ii^	0.95	2.75	3.401 (6)	126.
C12A—H12A···Cg4^ii^	0.95	2.70	3.394 (6)	130.
C5C—H5CA···Cg4^v^	0.95	2.94	3.691 (6)	137.
C11B—H11B···Cg5^i^	0.95	2.93	3.596 (6)	128.
C2A—H2AA···Cg6	0.95	2.83	3.425 (6)	122.

Symmetry codes: (i) *x*+1, *y*−1, *z*; (ii) *x*, *y*+1, *z*; (iii) *x*, *y*−1, *z*; (iv) *x*−1, *y*+1, *z*;  (v) *x*−1, *y*, *z*.
